# Mapping the Left Ventricular Summit for Ablation Success

**DOI:** 10.19102/icrm.2020.111204

**Published:** 2020-12-15

**Keywords:** Coronary sinus, catheter ablation, ventricular tachycardia

## Dr. Mehdirad comments

The stepwise approach to ventricular tachycardia (VT) originating from the left ventricular (LV) summit (LVS) described by Vyas et al. emphasizes the importance of conducting practical yet methodical mapping of the LVS structures and achieving successful ablation in the great cardiac vein (GCV).^1^

The majority of outflow tract (OT) arrhythmias originate from the anterior and superior septal aspects of the right ventricular (RV) OT (RVOT) with an underlying mechanism of triggered activity secondary to cyclic adenosine monophosphate–mediated delayed after-depolarizations.^2^ However, other origins, including the LV OT (LVOT), coronary cusps (CCs), and distal coronary sinus (CS)—especially when R/S transition occurs at V3 or earlier—have been described.^3,4^

Because of the absence of structural heart disease in OT VTs, a 12-lead electrocardiogram (ECG) is a useful modality by which to localize the area of VT/premature ventricular complex (PVC) origin. Although rare, OT VTs can be associated with subtle structural abnormalities (eg, aortic sinus of a valsalva aneurysm). The presence of multiple VT morphologies (either seen clinically or induced in the electrophysiology laboratory) or characterization as a re-entrant mechanism should raise concern that a complex arrhythmogenic substrate is present and a greater level of technical difficulty during ablation is more likely to be experienced.^5^

A 12-lead ECG in the case presented by Vyas et al. had the characteristics of VTs/PVCs originating from the GCV (an inferior axis and concordant R pattern in all precordial leads served as diagnostic markers for an LVOT origin in the surface ECG and suggested high ablation success via the GCV).^6^ Distal (DGCV) VTs/PVCs share the following ECG features: inferior axis, R pattern in all inferior leads, QS pattern in augmented vector left (aVL) and augmented vector right (aVR) leads, a dominant rs or rS pattern in lead I, a monophasic R or Rs pattern in all precordial leads, and a monophasic (positive concordance), transition occurring earlier than V1.^7^ The distinct ECG characteristics of VTs originating from the DGCV can help to differentiate VTs originating from adjacent LV endocardium sites of origin.^7^ In the presence of even subtle structural abnormalities, however, a 12-lead ECG may not be helpful for localizing the origin of VT/PVC as even subtle structural abnormalities may impact the typical ECG characteristics of VTs in normal hearts (e.g., RVOT or LVS).^5^

As a first step, an RV versus LV origin can be inferred by an early R-wave transition. VTs/PVCs originating from the RVOT feature a QRS transition by V4. Septal RVOT sites (left posteromedial aspect of the RVOT) exhibit narrower and taller R-waves in inferior leads, QRS without notching, and an S-wave in lead I.

Early QRS transition by V1–V2 and a lack of S-waves in V5–V6 are seen in the CC origin. Left CC (LCC) VTs/PVCs frequently exhibit a W- or M-pattern shape in V1. A downward notch in V1 has been suggested as an origin at the right CC–LCC commissure.^8^

In the case presented, RVOT activation mapping was not performed as a 12-lead ECG showed a concordant R-pattern in all precordial leads, which was a marker for an LVOT origin. As a result, LCC was mapped first where a good early signal was not found. In addition, the corresponding unipolar mapping signal showed an initial positive wave.

As expected, the GCV was the next structure indicated for mapping where, intuitively (based on 12-lead ECG characteristics), the earliest activation time was 30 ms earlier than the surface QRS with a distinct presystolic potential where ablation was successful. The fact that the patient was in ongoing hemodynamically stable sustained VT made both the mapping and ablation much easier to complete.

The LVS of the heart, nicknamed the “Bermuda Triangle” (BT) by some, is an area of the myocardium located between the following three neighboring structures: the posterior RVOT, the LCC, and the distal CS [ie, the origin of the GCV, the anterior interventricular vein (AIV)].^9^ This epicardial area is not easily accessible; the closer the focus of the VT/PVCs is to the center of the LVS, the more difficult it is to successfully locate the focus from any of the LVS borders. It is important to underscore that the RVOT is anterior and “leftward” to the aortic root.

Currently, several approaches to ablate VTs/PVCs originating from the LVS/BT through the RVOT (anteroseptal/posteroseptal) exist, including via the LCC, the LV myocardium just beneath the LCC, the distal CS (GCV, AIV), the septal perforator vein, and a percutaneous subxiphoid epicardial approach. Although these anatomical structures are electrophysiologically accessible, there is a small area of the myocardium adjacent to all of them that is not accessible. If ablation from conventional and commonly approached structures (ie, RVOT, LCC, GCV) fails, then the percutaneous subxiphoid epicardial approach may be considered as the next option. Although ablation through the left atrial appendage where the ablation electrode is directed toward the left LVS/BT may be considered, due to a high risk for perforation inherent with ablation from the left atrial appendage, only rare case reports on this approach have been published.^10^

In addition to intramural thrombosis and cardiac tamponade, as the LVS is an area surrounded by the left anterior descending (LAD) artery, the first septal perforating branch, and the left circumflex artery, the most serious complication involved with the ablation procedure is coronary artery injury. Integration of three-dimensional electroanatomical mapping with coronary angiography during an LVS ablation procedure is a new method with which real-time visualization of cardiac structures can be achieved.^11^

Ali Mehdirad, md (ali.mehdirad@va.gov)^1^

^1^Chief of Medicine, Carl Vinson VA Medical Center, Dublin, GA, USA

Dr. Mehdirad reports no conflicts of interest for the published content.

References1.VyasALokhandwalaYMahajanAWhich way to the summitJ Innov Cardiac Rhythm Manage202011124313431610.19102/icrm.2020.1101201PMC7769508334089512.MovsowitzCSchwartzmanDCallansDJIdiopathic right ventricular outflow tract tachycardia: narrowing the anatomic location for successful ablation.Am Heart J19961315930936[CrossRef][PubMed]861531210.1016/s0002-8703(96)90175-13.BetenskyBPParkREMarchlinskiFEThe V (2) transition ratio: a new electrocardiographic criterion for distinguishing left from right ventricular outflow tract tachycardia origin.J Am Coll Cardiol2011572222552262[CrossRef][PubMed]2161628610.1016/j.jacc.2011.01.0354.TannerHHindricksGSchirdewahnPOutflow tract tachycardia with R/S transition in lead V3: six different anatomic approaches for successful ablation.J Am Coll Cardiol2005453418423[CrossRef][PubMed]1568072210.1016/j.jacc.2004.10.0375.OlsonNFerreiraSWMikolajczakPCMehdiradAAortic sinus of valsalva aneurysm isolation by radiofrequency ablation in outflow tract ventricular tachycardia.World J Cardiovasc Dis20144413113710.4236/wjcd.2014.440196.BogossianHFrommeyerGNiniosISpot diagnosis of inferior axis and concordant R-pattern predicts left ventricular inflow tract tachycardia: ablation from the great cardiac vein of an underdiagnosed entity.Int J Cardiol2016214175179[CrossRef][PubMed]2706165510.1016/j.ijcard.2016.03.1837.LinYNXuJPanYQAn electrocardiographic sign of idiopathic ventricular tachycardia ablatable from the distal great cardiac vein.Heart Rhythm2020176905914[CrossRef][PubMed]3202804710.1016/j.hrthm.2020.01.0278.BalaRAblation of ventricular arrhythmias originating from the right coronary cusp–left coronary cusp commissure.J Innov Cardiac Rhythm Manage20111213814310.19102/icrm.2011.0201059.AltmannDKnechtSSticherlingCAmmannPOsswaldSKuhneMVentricular tachycardia originating from the “Bermuda Triangle”.Cardiovasc Med201316070820821010.4414/cvm.2013.0017010.YakubovASalayevOHamrayevRSultankhonovSA case of successful ablation of ventricular tachycardia focus in the left ventricular summit through the left atrial appendage: a case report.Eur Heart J: Case Rep201824yty110[CrossRef][PubMed]3102018610.1093/ehjcr/yty110PMC642603011.LiXLiJChuHLiuXA novel ablation strategy of premature ventricular contractions originating from summit guided by CartoUNIVU module.Clin Cardiol2020439963967[CrossRef][PubMed]3242830210.1002/clc.23390PMC7462194

## Dr. Hutchinson discusses

**Figure 1:**
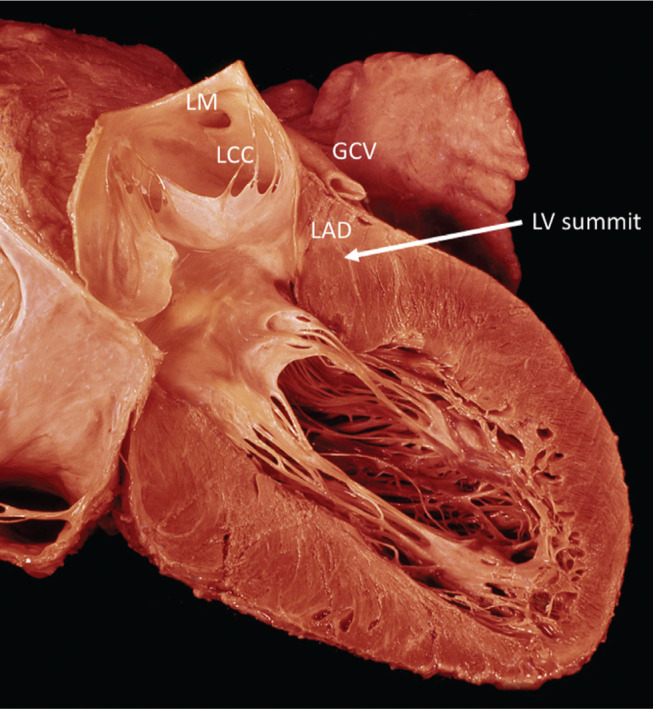
Pathologic section of the LV summit region. This region at the superior aspect of the left ventricular myocardium is bounded by the bifurcation of the LAD and left circumflex coronary arteries. One can appreciate from this specimen that the LCC and the GCV provide access to the superior aspect of the epicardial summit for catheter ablation. LAD: left anterior descending coronary artery; LM: left main coronary ostium. Illustration is from the Wallace A. McAlpine, MD collection, courtesy of the UCLA Cardiac Arrhythmia Center.

Vyas et al. report the case of a 57-year-old man with episodes of symptomatic wide complex tachycardia (WCT) who underwent catheter ablation.^1^ The approach to this patient should begin with a close examination of the spontaneous WCT morphology to generate a differential diagnosis. This includes aberrant supraventricular tachycardia, pre-excited tachycardia, and ventricular tachycardia (VT). In this case, we have a right-bundle, right-inferior-axis tachycardia with precordial concordance. Close examination of the patient’s sinus rhythm ECG (not shown in the case report) is necessary to confirm the presence of baseline conduction abnormalities or ventricular pre-excitation. The wide atypical right branch bundle block (RBBB) pattern shown would be an unusual pattern of aberration. Given the appearance of this patient’s WCT, one would anticipate early ventricular activation at the superior lateral mitral annulus. Anterogradely conducting accessory pathways in this anatomical location may often produce subtle degrees of pre-excitation due to competition with ventricular activation over the His–Purkinje system. If there is any question regarding the presence of pre-excitation, bedside vagal maneuvers or the administration of adenosine can readily unmask accessory pathway conduction. There is clear evidence of 1:1 atrioventricular (AV) association during the tachycardia presented in the upper panel of Vyas et al.’s **Figure 1**. The report is unclear whether this specific tracing was recorded during spontaneous tachycardia or (as I suspect) during atrial overdrive pacing of tachycardia. In any event, the demonstration of QRS fusion with atrial overdrive pacing during WCT strongly suggests VT as the mechanism (see Vyas et al.’s **Figure 1**, lower panel). The VT mechanism may be focal or reentrant and tachycardias involving the His–Purkinje system (eg, fascicular reentry) should be considered.

Although the morphology of this patient’s VT can certainly be present in normal hearts, it should also raise the specter of an occult arrhythmogenic cardiomyopathy. Further diagnostic testing may identify occult cardiomyopathic processes, particularly in patients with sustained VT. A prior study performed cardiovascular magnetic resonance (CMR) imaging in 46 consecutive patients presenting with presumed idiopathic ventricular arrhythmias and reported structural abnormalities in 41% of patients with tachycardias originating in the LV as compared with in 5% of patients with tachycardias originating from the RV.^2^ The presence of an arrhythmogenic cardiomyopathy poses important issues for ablation planning in addition to its potential implications for subsequent sudden death risk stratification. A prior study used CMR imaging to characterize substrate pattern in 19 patients with nonischemic LV cardiomyopathy and observed predominant inferior and lateral involvement in 47% of cases.^3^ Epicardial ablation was required to eliminate VT in five of the eight patients with inferior and lateral substrate patterns. Pacemapping from the basal superior LV epicardium produces an initial Q-wave in limb lead 1 and, thus, the presence of this finding during spontaneous VT may suggest the need for a nonendocardial ablation approach.^4^ Appropriate patient education is also essential for cases in which epicardial mapping may be required.

One should approach the mapping of ventricular arrhythmias in a manner that facilitates sampling of all potentially relevant areas. Presuming that this patient has idiopathic VT, it seems to be originating near the superior lateral mitral valve annulus. Thus, we should anticipate accessing the aortic sinuses, the LV endocardium, and the epicardium via both the coronary venous system and, if needed, via a pericardial-access approach. Dedicated arterial access is required for aortic root mapping and can often facilitate easier access to the superior and lateral aspects of the mitral annulus than when using a transseptal approach. One should place a multipolar catheter into the distal CS, ideally with the distal poles located within the great cardiac vein. For summit PVCs with a left bundle morphology, mapping of the AIV may be required. Using smaller diagnostic catheters may facilitate sampling of these distal regions. Given the possibility of arrhythmias involving the Purkinje system, placing a dedicated catheter in the His position may be useful. Standard patient-related and perioperative considerations for pericardial access should also be considered.

As discussed already, the presence of a QS complex in lead 1 of the spontaneous WCT is not commonly seen with endocardial pacemaps from this region; thus, we should anticipate the earliest activation to occur closer to the epicardium. As such, it is not surprising that activation times obtained from the left CS and the LV endocardium were not presystolic in this case. The LVS encompasses a triangular section of the LV epicardium subtended by the LAD coronary artery septally and the circumflex coronary artery basally. The summit is divided by the course of the coronary veins into inaccessible and accessible regions. The latter is more relevant for the current patient given the ECG morphology.^5^

The general approach in summit arrhythmias is to find the safest proximate site to the target. This requires understanding the relevant anatomical relationships vis-à-vis the coronary vasculature. Adopting the multipolar CS catheter to bracket the earliest annular region is extremely useful. Failure to pass the multipolar catheter distally is usually due to a tight Vieussens valve, which can be overcome with a dedicated long introducer and guidewire. The information provided by the CS recordings is essential to have in these cases and time spent to gather such is well worth the effort. Occasionally, the coronary vein may provide an excellent ablation target on the basis of activation and pacemapping, thus obviating the need for endocardial access at all. The current case presentation highlights the electrogram characteristics that suggest proximity of the CS recording site to the VT site of origin. Note the steep QS complex on the local unipolar electrogram and the presence of a consistent early sharp potential on the bipolar channel that precedes the unipolar onset. Whether this is a local CS muscular potential is debatable. It would be nice to have seen a pacemap from the earliest CS site; however, an imperfect match would not necessarily predict ablation failure. Coronary angiography is essential to determine their proximity of the desired CS ablation site. Ablation within the CS is challenging due to rapid impedance drops that limit conventional energy titration schema. When CS ablation is not possible due to the proximity to the coronary arteries, specific ECG criteria may predict success with a percutaneous epicardial approach.^6^

When the activation and pacemapping within the CS are less perfect, then sampling of the LV endocardium is completed. The CS catheter provides an excellent fluoroscopic or electroanatomical reference to begin targeted endocardial mapping. When the CS and LV endocardium exhibit similar activation times, this often suggests an intramural focus and ablation ideally should be initiated in the endocardium. In these cases, the use of longer lesions with unipolar ablation is often necessary to suppress the arrhythmia. The use of simultaneous unipolar or bipolar ablation in this region can be considered in refractory cases; however, data supporting safety and efficacy are limited at present.^7^ Alcohol instillation is another consideration; however, its’ use is better studied in arrhythmias originating within the septum in which small branches can be subselected to limit collateral damage.^8^

Overall, this case highlights the importance of using a systematic approach in evaluating WCTs. One should consider pursuing adjunctive diagnostic testing in evaluating occult cardiomyopathic processes as their presence may increase the level of procedural complexity. Arrhythmias originating from the LV summit pose significant ablation challenges due to the anatomical complexity and proximity to critical structures inherent with this location. CS mapping is of critical importance in targeting summit arrhythmias as it can provide activation and pacemapping data from an epicardial location and, in selected cases, an attractive site for ablation.

Mathew D. Hutchinson, md, facc, fhrs (mathewhutchinson@shc.arizona.edu)^1^

^1^Sarver Heart Center, University of Arizona College of Medicine Tucson, Tucson, AZ, USA

Dr. Hutchinson reports no conflicts of interest for the published content.

References1.VyasALokhandwalaYMahajanAWhich way to the summitJ Innov Cardiac Rhythm Manage202011124313431610.19102/icrm.2020.1101201PMC7769508334089512.NuciforaGMuserDMasciPGPrevalence and prognostic value of concealed structural abnormalities in patients with apparently idiopathic ventricular arrhythmias of left versus right ventricular origin: a magnetic resonance imaging study.Circ Arrhythm Electrophysiol201473456462[CrossRef][PubMed]2477154310.1161/CIRCEP.113.0011723.PiersSRTaoQvan Huls van TaxisCFSchalijMJvan der GeestRJZeppenfeldKContrast-enhanced MRI-derived scar patterns and associated ventricular tachycardias in nonischemic cardiomyopathy: implications for the ablation strategy.Circ Arrhythm Electrophysiol201365875883[CrossRef][PubMed]2403613410.1161/CIRCEP.113.0005374.BazanVGerstenfeldEPGarciaFCSite-specific twelve-lead ECG features to identify an epicardial origin for left ventricular tachycardia in the absence of myocardial infarction.Heart Rhythm200741114031410[CrossRef][PubMed]1795439910.1016/j.hrthm.2007.07.0045.YamadaTMcElderryHTDoppalapudiHIdiopathic ventricular arrhythmias originating from the left ventricular summit: anatomic concepts relevant to ablation.Circ Arrhythm Electrophysiol201036616623[CrossRef][PubMed]2085537410.1161/CIRCEP.110.9397446.SantangeliPMarchlinskiFEZadoESPercutaneous epicardial ablation of ventricular arrhythmias arising from the left ventricular summit: outcomes and electrocardiogram correlates of success.Circ Arrhythm Electrophysiol2015823373943[CrossRef][PubMed]2563759610.1161/CIRCEP.114.0023777.FutymaPSanderJCiapalaKBipolar radiofrequency ablation delivered from coronary veins and adjacent endocardium for treatment of refractory left ventricular summit arrhythmias.J Interv Card Electr2020583307313[CrossRef][PubMed]10.1007/s10840-019-00609-9314024158.KreidiehBRodriguez-ManeroMSchurmannPIbarra-CortezSHDaveASValderrabanoMRetrograde coronary venous ethanol infusion for ablation of refractory ventricular tachycardia.Circu Arrhythm Electrophysiol201697e004352[CrossRef][PubMed]10.1161/CIRCEP.116.004352PMC505310227406606

## Dr. Gerstenfeld explains

Vyas et al. present an interesting case of a 57-year-old male with sustained idiopathic VT originating from the region of the LVS, treated successfully with catheter ablation.^1^ The patient presented with monomorphic VT requiring electrical cardioversion. Echocardiogram and coronary angiogram findings were normal, suggesting the VT was idiopathic (ie, not associated with scar or structural heart disease). Sustained monomorphic VT from the LVS in a structurally normal heart is uncommon. Depending on the availability of more advanced imaging modalities, one might consider performing CMR imaging with delayed enhancement (DE-MRI) in such a patient. Subtle scarring not detected by echocardiography might be detected by DE-MRI and could portend a worse prognosis. We don’t have an example of a sinus rhythm 12-lead ECG or chest X-ray in this case; both would be important to examine. Any evidence of conduction disease and/or hilar adenopathy could also suggest a diagnosis of cardiac sarcoidosis, which would merit a cardiac positron-emission tomography (PET) scan to identify active inflammation. While unlikely, the treatment of cardiac sarcoidosis would be quite different from that for idiopathic VT.

In this case, the authors proceeded to perform catheter ablation without first trying antiarrhythmic therapy. I think that this approach is perfectly reasonable. Electrophysiologic study has the advantage of aiding in the diagnosis by excluding scar with voltage mapping and potentially curing the VT in a young man with an apparently normal heart and is considered guideline-directed first-line therapy.^2^ The 12-lead ECG in this case has a morphology that is clearly VT, with a monophasic R in V1, positive concordance across the precordium, and a QS pattern in lead I. There is also an interesting cycle-length/morphology QRS alternans, which can sometimes occur at rapid ventricular rates. The morphology clearly places the VT exit at the superior mitral annular region and the QS in lead I should raise the suspicion of an epicardial exit. This region at the superior aspect of the LV septum has been termed the LV “summit” and can pose unique challenges for catheter ablation. The LVS is defined as the superior most aspect of the LV septum, bounded by the bifurcation of the LAD and circumflex coronary arteries **(Figure 1)**. The LVS contains an inferior and lateral portion that is often accessible by catheter ablation from the LV endocardium together with a superior “inaccessible” portion that is more challenging to reach and which may require more advanced approaches.

**Figure 2:**
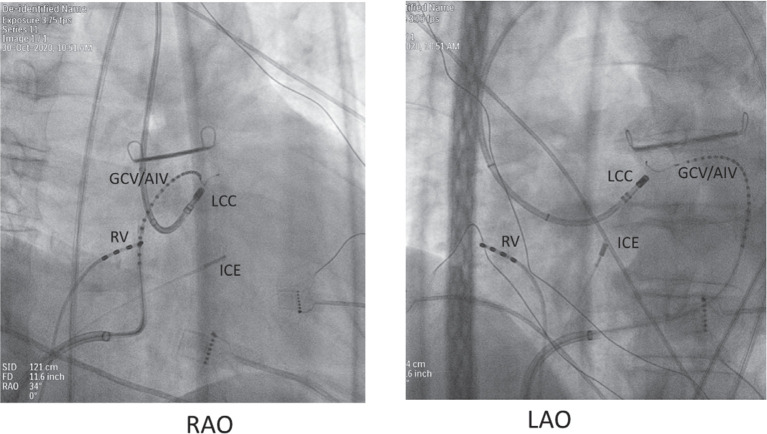
Right anterior oblique (RAO, left panel) and left anterior oblique (LAO, right panel) fluoroscopic views in a patient undergoing ventricular tachycardia ablation. A 20-electrode microcatheter is advanced via the CS to the GCV/AIV region. An ablation catheter is shown with its tip in the left coronary cusp, demonstrating the proximity of the left coronary cusp to the distal GCV. ICE: intracardiac echocardiography catheter; RV: right ventricular catheter.

When approaching an LVS VT, we will typically start with a CS venogram and then position a small multipolar microcatheter out the distal great coronary vein (GCV) into the AIV to facilitate detailed mapping **(Figure 2)**. Although these branches are often too small in caliber to allow an ablation catheter to enter, they do allow one to approach adjacent structures for catheter ablation, which may be efficacious. Examination of the 12-lead ECG is also helpful. Abularach et al.^3^ found that, when the aVL/aVR q-wave ratio was less than 1.45, the PVC/VT could often be ablated from the left sinus of Valsalva as opposed to the GCV. In this case, the aVL/aVR ratio (while difficult to measure on paper) appears to be greater than 1.45, suggesting that ablation from the GCV might be necessary.

The authors appropriately began mapping in the left sinus of Valsalva (LSV) and found a bipolar electrogram that is only slightly pre-QRS, with a small r-wave on the unipolar recording suggesting exit from a site deep or adjacent to the catheter tip. The ablation catheter tip in the authors’ **Figure 2** appears to show the ablation catheter in the aortic root, interpreted as the left sinus of Valsalva. In their **Figure 3**, the authors advance a 7-French ablation catheter out of the GCV, where they find an earlier signal and QS unipolar signal that results in successful ablation.

Several aspects of this case are worthy of discussion. Whenever possible, we always prefer ablation from the aortic sinus over the GCV because of (1) the ability to deliver higher power and (2) greater distance from the epicardial coronary arteries. Certainly, the ECG and activation in the LSV suggest that ablation may not be effective in this case. However, the use of intracardiac echocardiography, when available, can facilitate confirmation of location and mapping in the aortic sinuses, which often need to be extensively mapped as a separate cardiac chamber. If activation in the GCV and LSV are similar or even if the GCV is slightly earlier than the LSV, we would usually start with ablation in the LSV for the reasons stated. If ineffective, mapping of adjacent sites in the LV or RV endocardium using the earliest electrode on the multipolar microcatheter as a guide is undertaken, which may yield an effective site for ablation. If none of these sites are suitable, then manipulation of the ablation catheter into the GCV is reasonable. A CS venogram can help to determine the various branches and whether they will accommodate a radiofrequency ablation catheter or not. Irrigated ablation is almost always required in the GCV given its small caliber. Vyas et al. describe using a 7-French irrigated ablation catheter, which is an advantage in their particular case as the smallest irrigated ablation catheter available in the United States is on an 8-French shaft, which may not allow mapping into small venous branches.

As the authors have noted, it is imperative to perform coronary angiography before ablating in the GCV, as the coronary veins often run in parallel with the arterial system. This is illustrated in the authors’ **Figure 3**, which demonstrates the catheter tip quite close to the LAD coronary artery. The authors ablated with 20 W of power in this location, which exemplifies the power limitations that exist when ablating inside a small vein due to low blood flow. Sometimes, manually increasing the catheter irrigation rate can permit higher delivered power. Would I have ablated at this location? It is always difficult to ascertain catheter location on still images as compared to examining moving cine views from multiple angles during a live case. Typically, the catheter tip should be located greater than 5 mm from a coronary artery to avoid injury, although this distance is somewhat empiric. In this case, I would certainly have heightened concern. Assuming the catheter tip electrode is 4 mm in diameter, it appears that it may be within 5 mm of a very proximal LAD and the tip is pointed directly at the artery, which will maximize heating. Damage to this artery could have catastrophic complications. If ablation were to be undertaken, I would certainly ask for an interventional colleague to be standing by with images of the artery taken before and after each ablation. In the end, one must weigh the benefits of successful ablation against the risk of complications. In this patient who presented with sustained monomorphic VT, an aggressive approach is certainly warranted. However, I would explore all feasible LV locations, as described above, before ablating at this location. Epicardial access has also been described for mapping and ablating LVOT arrhythmias, particularly since the exit of this VT appears epicardial by ECG. We typically try to avoid routine epicardial access in LVOT arrhythmias because of proximity to the coronary arteries and a thick layer of epicardial fat in this location, which often precludes adequate heating of the myocardium. Nevertheless, epicardial mapping can sometimes add to success in this location. Other options that can be considered are use of a half-normal saline irrigant from the earliest site in the LV endocardium to improve the lesion depth,^4^ coronary wire mapping/ablation via the LAD,^5^ or cryocatheter ablation in the GCV (which may be safer because the warm blood protects the LAD in this context).

I congratulate the authors for their successful ablation of a difficult arrhythmia. Such cases emphasize the importance of understanding the complex anatomy of the LVS the biophysics of ablation, and the expertise of multipolar venous mapping.

Edward P Gerstenfeld, md (Edward.Gerstenfeld@ucsf.edu)^1^

^1^Section of Cardiac Electrophysiology, Department of Medicine, University of California, San Francisco, CA, USA

Dr. Gerstenfeld reports no conflicts of interest for the published content.

References1.VyasALokhandwalaYMahajanAWhich way to the summitJ Innov Cardiac Rhythm Manage202011124313431610.19102/icrm.2020.1101201PMC7769508334089512.Al KhatibSStevensonWGAckermanMJ2017 AHA/ACC/HRS guideline for management of patients with ventricular arrhythmias and the prevention of sudden cardiac death.Heart Rhythm.20181510e73e18910.1016/j.hrthm.2017.10.036290973193.AbalarachMEJCamposBParkKMAblation of ventricular arrhythmias arising near the anterior epicardial veins from the left sinus of Valsalva region: ECG features, anatomic distance, and outcome.Heart Rhythm.201296865873[CrossRef][PubMed]2230661810.1016/j.hrthm.2012.01.0224.NguyenDTTzouWSSandhuAProspective multicenter experience with cooled radiofrequency ablation using high impedance irrigant to target deep myocardial substrate refractory to standard ablationJACC Clin Electrophysiol20184911761185[CrossRef][PubMed]3023639110.1016/j.jacep.2018.06.0215.RomeroJDiazJCHayaseJIntramyocardial radiofrequency ablation of ventricular arrhythmias using intracoronary wire mapping and a coronary reentry system: description of a novel technique.HeartRhythm Case Rep.201847285292[CrossRef][PubMed]3002327310.1016/j.hrcr.2018.03.005PMC6050428
